# Supplementary material for: Estimation of the morbidity and mortality of congenital Chagas disease: A systematic review and meta-analysis
Source: PLoS Negl Trop Dis. 2022 Nov 7;16(11):e0010376. doi: 10.1371/journal.pntd.0010376 (PMC9671465; doi:10.1371/journal.pntd.0010376)
Supplement: S1 File — (DOCX) [file pntd.0010376.s001.docx]

**S1 File**. Morbidity clinical signs of congenital Chagas disease

- hepatomegaly
- splenomegaly
- respiratory distress syndrome
- neurologic signs (not including convulsions)
- anasarca
- petechiae
- low Apgar score (<7 at 1 minute)
- abnormal electrocardiographic findings
- anemia
- meningoencephalitis
- myocarditis
- congestive heart failure
- lesions in the digestive and/or central nervous system
- parasites in various tissues
- subependymal hemorrhage
- cardiomegaly
- premature rupture of the membrane (PROM)
- preterm birth
- low birth weight
- intra-uterine growth restriction
- small for gestational age
- neonatal intensive care unit (NICU) admission
