# Supplementary material for: Estimation of the morbidity and mortality of congenital Chagas disease: A systematic review and meta-analysis
Source: PLoS Negl Trop Dis. 2022 Nov 7;16(11):e0010376. doi: 10.1371/journal.pntd.0010376 (PMC9671465; doi:10.1371/journal.pntd.0010376)
Supplement: S5 File — (DOCX) [file pntd.0010376.s005.docx]

**S5 File.** Risk of bias algorithms, summary within-domain risk of bias, and results

**Algorithm**

EXPOSURE = congenital Chagas disease diagnosed by gold standard method

OUTCOME = clinical signs of Chagas infection

*Exposure assessed through gold standard method*

The gold standard for diagnosis of congenital Chagas disease is parasitological examination using the microhematocrit or microstrout testing methods. This test should be performed at birth, and if negative the test should be repeated again one month later. Serological tests should be performed if there was no previous screening of the neonate or the parasitological and/or molecular test results were negative. Serological assays can be performed at 10 months of age, when maternal antibodies have waned in the infant [1].

*Was loss to follow-up after baseline 20% or less?*

- 20% or less **LOW** risk of bias
- 21% - 30% **PARTIAL** risk of bias
- 31% or more **HIGH** risk of bias

*Descriptive data: characteristics of study participants (e.g. demographic, clinical, social) and information on exposure, indicate the number of participants with missing data for each variable of
interest, summarize follow-up time (e.g. average and total amount)*

- Characteristics = those that are outlined in our data extraction form.
  - Data extraction form -
    - Maternal characteristics:
      - Participants Nationality
      - Current Residence (Urban/Rural/Mixed)
      - Participants Socioeconomic Status
      - Other Reported Characteristics
      - Number of Chagas cases
      - Diagnostic Test Used
      - Diagnostic Sample Used
      - Timing of Diagnostic
    - Congenital case characteristics:
      - Study Participant Characteristics
      - Birth Complications (Not Chagas Related)
  - If study includes mothers and infants
    - 7-10 **LOW** risk of bias
    - 4-6 **PARTIAL** risk of bias
    - 0-3 **HIGH** risk of bias
  - If study includes only infants
    - 2 **LOW** risk of bias
    - 1 **PARTIAL** risk of bias
    - 0 **HIGH** risk of bias

Scenarios where N/A was used:

- Methods for measuring exposure and outcome variables:
  - Was loss to follow-up after baseline 20% or less?
    - In retrospective case-control and cross-sectional studies, this was counted as N/A
- Methods to control confounding:
  - Only used in scenarios where measures of association were calculated
- Statistical methods:
  - Describe all statistical methods, including those used to control for confounding
    - If confounding was not measured, and studies were only reporting crude descriptive statistics, this was marked as N/A
  - *Cohort study—Explain how loss to follow-up was addressed
    Case-control study—Explain how matching of cases and controls was addressed
    Cross-sectional study—Describe analytical methods taking account of sampling strategy*
    - If there was no loss to follow-up
    - If there was no matching

**Summary within-domain risk of bias**

|  | **Methods for selecting study participants** | **Methods for measuring exposure and outcome variables** | **Methods to control confounding** | **Reporting of results** | **Statistical methods** | **Declaration of conflict and ethical statements** |
| --- | --- | --- | --- | --- | --- | --- |
| **Apt-2013 [2]** | / | + |  | / | + | + |
| **Arcavi-1993 [3]** | + | / |  | + | - | - |
| **Azogue-1991 [4]** | + | + |  | / |  | / |
| **Bahamonde-2002 [5]** | + | + |  | / | - | - |
| **Barona-Vilar-2012 [6]** | + | + |  | / | + | + |
| **Barousse-1978 [7]** | + | / |  | / | / | / |
| **Basile-2019 [8]** | + | / | + | + | / | + |
| **Bern-2009 [9]** | + | / |  | / | / | + |
| **Bisio-2011 [10]** | + | / |  | / | / | + |
| **Bittencourt-1985 [11]** | / | / |  | + | - | - |
| **Buekens-2018 [12]** | + | + |  | + | / | + |
| **Cardoso-2012 [13]** | + | / |  | / | / | + |
| **Castillo-1984 [14]** | / | - |  | - | - | / |
| **Contreras-1999 [15]** | / | / |  | - | - | - |
| **Cucunuba-2012 [16]** | / | - |  | / | - | - |
| **De Rissio-2010 [17]** | + | / |  | + | + | + |
| **Florez-Chavez-2011 [18]** | / | / |  | / | + | / |
| **Francisco-González-2019 [19]** | / | + |  | / | + | + |
| **Freilij-1995 [20]** | / | / |  | / | + | - |
| **Fumado-2014 [21]** | + | / |  | / | - | - |
| **Giménez-2010 [22]** | / | / |  | / | - | - |
| **Iglesias-1985 [23]** | / | - |  | / | - | - |
| **Mallimaci-2010 [24]** | / | + |  | + | + | + |
| **Martínez de Tejada-2009 [25]** | + | / |  | / | - | - |
| **Mayer-2010 [26]** | + | + |  | / | + | + |
| **Mendoza-2014 [27]** | / | + |  | / | - | - |
| **Mendoza-1983 [28]** | + | / |  | + |  | / |
| **Messenger-2017 [29]** | + | / | + | + | + | + |
| **Munoz-2009 [30]** | + | + | + | + | / | + |
| **Munoz-1982 [31]** | + | + |  | / | - | / |
| **Murcia-2017 [32]** | / | / |  | - | + | + |
| **Nisida-1999 [33]** | / | / |  | / | - | / |
| **Ortiz-2012 [34]** | - | / |  | / | - | + |
| **Otero-2012 [35]** | + | + |  | + |  | / |
| **Rodari-2018 [36]** | / | + |  | / | / | / |
| **Rubio-1962 [37]** | - | / |  | / | - | - |
| **Salas-2007 [38]** | + | - | + | / | / | + |
| **Sasagawa-2015 [39]** | / | + |  | + | / | + |
| **Sosa-Estani-2009 [40]** | + | / |  | / | + | + |
| **Streiger-1995 [41]** | + | / |  | / | - | / |
| **Tello-1982 [42]** | + | / |  | / | - | / |
| **Torrico-2004 [43]** | / | / |  | - | + | + |
| **Valenzuela-1984 [44]** | / | - |  | / |  | / |
| **Valperga-1992 [45]** | / | + |  | + |  | - |
| **Vicco-2016 [46]** | / | + |  | + | / | + |
| **Villablanca-1984 [47]** | / | - |  | / |  | / |
| **Zaidenberg-1993 [48]** | / | / |  | / | - | - |

**Results**

Within domain 1, 23(47.9%) articles had low risk of bias, 22(45.8%) had moderate, and 2(4.2%) had high risk. In Domain 2, 16(33.3%), 25(52.1%), and 6(12.5%) articles had low, moderate, and high risks of bias, respectively. Domain 3 had 4(8.3%) articles with low risk of bias; the remaining 43 articles were non-applicable within this domain. Domain 4 had 13(27.1%), 30(62.5%), and 4(8.3%) articles with low, moderate, and high risks of bias, respectively. Domain 5 had 12(25.0%) low, 11(22.9%) moderate, and 18(37.5%) high risks of bias; the remaining 6 articles were non-applicable. Domain 6 had 20(41.7%) articles with low risk of bias, 13(27.1%) moderate, and 14(29.2%) high. **Fig 5** summarizes the within-domain risk of bias assessment. Overall, 34(72.3%) articles were determined to have high risk of bias, 10(21.3%) were moderate, and 3(6.4%) were low.

**Fig 5. Within-domain Risk of Bias Summary**

**
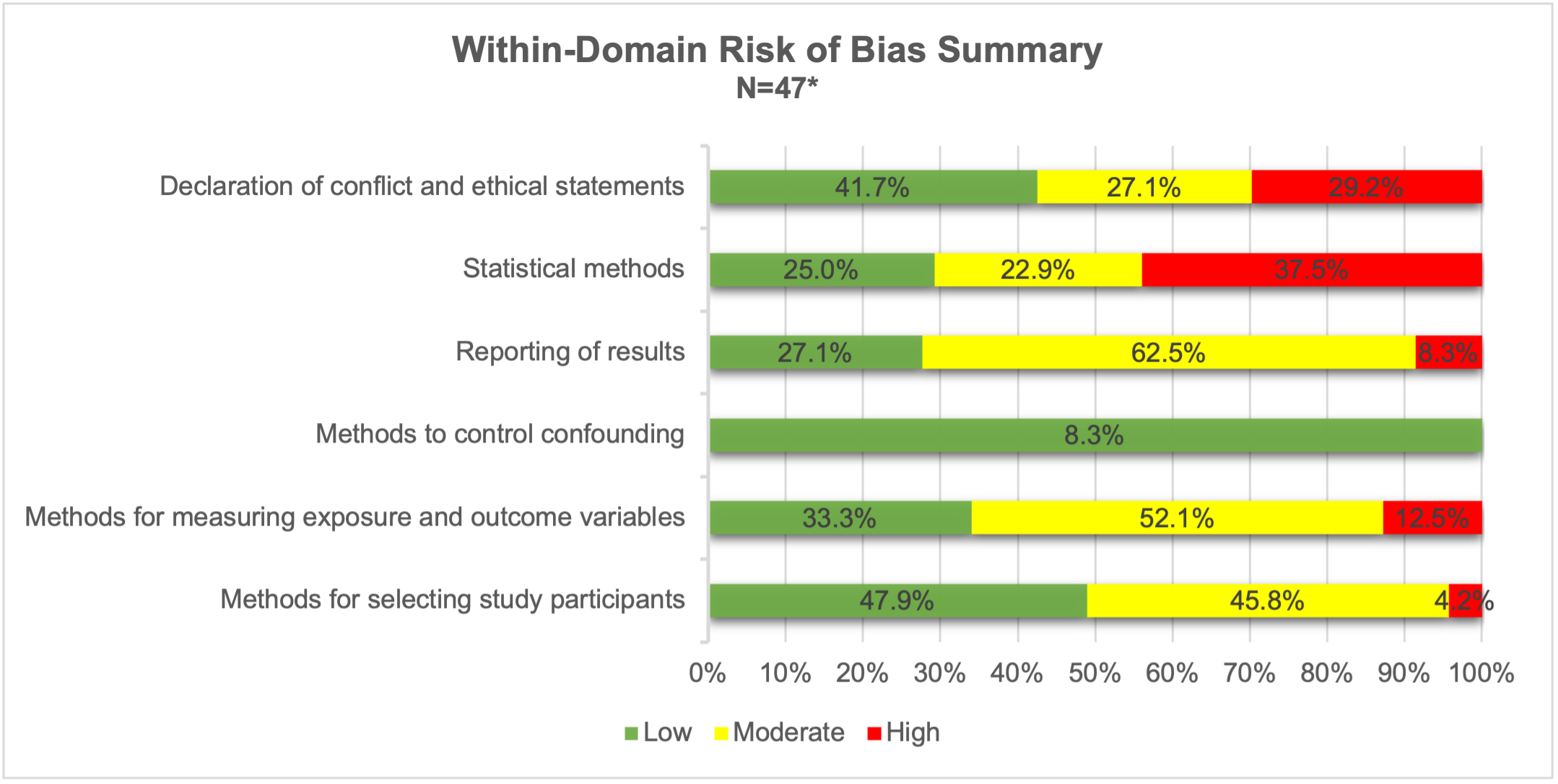
**

**^*^**Does not include percentage of N/A values within domains therefore totals for each category will not equate to 100%

**References**

1. Carlier Y, Altcheh J, Angheben A, Freilij H, Luquetti AO, Schijman AG, et al. Congenital Chagas disease: Updated recommendations for prevention, diagnosis, treatment, and follow-up of newborns and siblings, girls, women of childbearing age, and pregnant women. PLoS Negl Trop Dis. 2019;13(10):e0007694.

2. Apt W, Zulantay I, Arnello M, Oddó D, González S, Rodríguez J, et al. Congenital infection by *Trypanosoma cruzi* in an endemic area of Chile: a multidisciplinary study. Transactions of the Royal Society of Tropical Medicine and Hygiene. 2013;107(2):98-104.

3. Arcavi M, Orfus G, Griemberg G. Incidence of Chagas infection in pregnant women and newborn infants in a non-endemic area. Medicina. 1993;53(3):217-22.

4. Azogue E, Darras C. Prospective study of Chagas disease in newborn children with placental infection caused by Trypanosoma cruzi (Santa Cruz-Bolivia). Rev Soc Bras Med Trop [Internet]. 1991 [cited 2022 Oct 24];24(2):105–9. Available from: https://pubmed.ncbi.nlm.nih.gov/1841425/

5. Bahamonde MI, Baeza M, Chambel C, Ramirez C, Goycolea M, Cáceres J. Prevalencia de la infección transplacentaria por *Trypanosoma cruzi* en el Hospital de Calama, II Región-Chile. Revista de Patologia Tropical/Journal of Tropical Pathology. 2002;31(1):87-96.

6. Barona-Vilar C, Gimenez-Marti MJ, Fraile T, Gonzalez-Steinbauer C, Parada C, Gil-Brusola A, et al. Prevalence of *Trypanosoma cruzi* infection in pregnant Latin American women and congenital transmission rate in a non-endemic area: the experience of the Valencian Health Programme (Spain). Epidemiol Infect. 2012;140(10):1896-903.

7. Barousse A, Eposto M, Mandel S, Martínez F. Congenital Chagas' disease in a non-endemic area. Medicina. 1978;38(6 Pt 1):611-5.

8. Basile L, Ciruela P, Requena-Méndez A, Vidal MJ, Dopico E, Martín-Nalda A, et al. Epidemiology of congenital Chagas disease 6 years after implementation of a public health surveillance system, Catalonia, 2010 to 2015. Eurosurveillance. 2019;24(26):1900011.

9. Bern C, Verastegui M, Gilman RH, Lafuente C, Galdos-Cardenas G, Calderon M, et al. Congenital *Trypanosoma cruzi* transmission in Santa Cruz, Bolivia. Clin Infect Dis. 2009;49(11):1667-74.

10. Bisio M, Seidenstein ME, Burgos JM, Ballering G, Risso M, Pontoriero R, et al. Urbanization of congenital transmission of *Trypanosoma cruzi*: prospective polymerase chain reaction study in pregnancy. Trans R Soc Trop Med Hyg. 2011;105(10):543-9.

11. Bittencourt ACL, Mota E, Ribeiro Filho R, Fernandes LG, Almeida PRCd, Sherlock ÍRdA, et al. Incidence of congenital Chagas' disease in Bahia, Brazil. 1985.

12. Buekens P, Cafferata ML, Alger J, Althabe F, Belizán JM, Bustamante N, et al. Congenital transmission of *Trypanosoma cruzi* in Argentina, Honduras, and Mexico: an observational prospective study. The American Journal of Tropical Medicine and Hygiene. 2018;98(2):478-85.

13. Cardoso EJ, Valdéz GC, Campos AC, de la Luz Sanchez R, Mendoza CR, Hernández AP, et al. Maternal fetal transmission of *Trypanosoma cruzi*: a problem of public health little studied in Mexico. Experimental Parasitology. 2012;131(4):425-32.

14. Castillo S, Mardones C, Hormazábal G, Cubillos R, Barahona N, Zepeda S, et al. Chagas' disease in Chile. Urban sectors. VI. Frequency of Chagas' infection in blood donors and in mothers and newborn infants of the cities of Antofagasta and Calama. II Region (1983-1984). Boletin Chileno de Parasitologia. 1984;39(1-2):28-32.

15. Contreras S, Fernández MR, Agüero F, Desse Desse J, Orduna T, Martino O. Congenital Chagas-Mazza disease in Salta, Argentina. Rev Soc Bras Med Trop. 1999;32(6):633-6.

16. Cucunuba Z, Valencia C, Flórez C, León C, Castellanos Y, Cardenas A, et al. Pilot program for surveillance of congenital Chagas disease in Colombia 2010-2011. International Journal of Infectious Diseases. 2012;16:e343.

17. De Rissio AM, Riarte AR, Garcia MM, Esteva MI, Quaglino M, Ruiz AM. Congenital *Trypanosoma cruzi* infection. Efficacy of its monitoring in an urban reference health center in a non-endemic area of Argentina. Am J Trop Med Hyg. 2010;82(5):838-45.

18. Flores-Chavez MD, Merino FJ, García-Bujalance S, Martin-Rabadan P, Merino P, Garcia-Bermejo I, et al. Surveillance of Chagas disease in pregnant women in Madrid, Spain, from 2008 to 2010. Euro Surveill. 2011;16(38).

19. Francisco-González L, Gastañaga-Holguera T, Montero BJ, Pérez ZD, Ramos MI, Amador PM, et al. Seroprevalence and vertical transmission of Chagas disease in a cohort of Latin-American pregnant women in a tertiary hospital in Madrid. Anales de Pediatría (English Edition). 2018;88(3):122-6.

20. Freilij H, Altcheh J. Congenital Chagas' disease: diagnostic and clinical aspects. Clinical Infectious Diseases. 1995;21(3):551-5.

21. Fumado V, Juncosa T, Posada E, Fisa R, Gallego M, Gascon J. Paediatric Chagas in a non-endemic area. Enfermedades infecciosas y microbiologia clinica. 2014;32(5):293-6.

22. Giménez MJ, Gómez-Ruiz MD, Calabuig A, Perez-Tamarit A, Otero MC, Fernández-Silveira J, et al., editors. Congenital transmission of Chagas’ disease in Latin American immigrants in a health department of Valencia, Spain. European Congress of Clinical Microbiology and Infectious Diseases; 2010; Vienna, AT: Clinical Microbiology and Infection.

23. Iglesias J, Schenone S, Contreras M, Danitz A, Pineda C, Badulli A, et al. Chagas' disease in Chile. Urban sections. IX. Frequency of Chagas' disease in mothers and newborn infants of the Eastern Section of the Metropolitan area, Chile, 1985. Boletin chileno de parasitologia. 1985;40(1-2):30-3.

24. Mallimaci MC, Sosa-Estani S, Russomando G, Sánchez Z, Sijvarger C, Alvarez IM, et al. Early diagnosis of congenital *Trypanosoma cruzi* infection, using shed acute phase antigen, in Ushuaia, Tierra del Fuego, Argentina. Am J Trop Med Hyg. 2010;82(1):55-9.

25. Martínez de Tejada B, Jackson Y, Paccolat C, Irion O. Congenital Chagas disease in Geneva: diagnostic and clinical aspects. Revue Medicale Suisse. 2009;5(222):2091-2, 4.

26. Mayer JP, Biancardi M, Altcheh J, Freilij H, Weinke T, Liesenfeld O. Congenital infections with *Trypanosoma cruzi* or Toxoplasma gondii are associated with decreased serum concentrations of interferon-c and interleukin-18 but increased concentrations of interleukin-10. Annals of Tropical Medicine & Parasitology. 2010;104(6):485-92.

27. Mendoza C, Ruiz S, Maya S, Del Río M, Rodríguez E. Vertical transmission of *T. cruzi*: Our experience at a secondary hospital in Barcelona. The Journal of Maternal-Fetal & Neonatal Medicine. 2014;27(sup1):1-437.

28. Mendoza J, Longa E, Contreras M, Sandoval L, Amigo C. Chagas' disease in Chile. Urban sections. III. Frequency of Chagasic infection in mothers and newborns from the Hospital of Copiapo (III Region, Chile). Boletin Chileno de parasitologia. 1983.

29. Messenger LA, Gilman RH, Verastegui M, Galdos-Cardenas G, Sanchez G, Valencia E, et al. Toward improving early diagnosis of congenital Chagas disease in an endemic setting. Clin Infect Dis. 2017;65(2):268-75.

30. Munoz J, Coll O, Juncosa T, Verges M, del Pino M, Fumado V, et al. Prevalence and vertical transmission of *Trypanosoma cruzi* infection among pregnant Latin American women attending 2 maternity clinics in Barcelona, Spain. Clin Infect Dis. 2009;48(12):1736-40.

31. Munoz P, Lorca M, Thiermann E, Astorga B, Arias A, Pino S. Transmisión congénita del *Trypanosoma cruz*i: Investigación en la maternidad del Hospital San Juan de Dios, de Santiago. Revista Chilena de Pediatría. 1982;53(1-6):22-7.

32. Murcia L, Simón M, Carrilero B, Roig M, Segovia M. Treatment of infected women of childbearing age prevents congenital *Trypanosoma cruzi* infection by eliminating the parasitemia detected by PCR. The Journal of infectious diseases. 2017;215(9):1452-8.

33. Nisida IVV, Amato Neto V, Braz LMA, Duarte MIS, Umezawa ES. A survey of congenital Chagas’ disease, carried out at three Health Institutions in São Paulo City, Brazil. Revista do Instituto de Medicina Tropical de São Paulo. 1999;41(5):305-11.

34. Ortiz S, Zulantay I, Solari A, Bisio M, Schijman A, Carlier Y, et al. Presence of *Trypanosoma cruzi* in pregnant women and typing of lineages in congenital cases. Acta tropica. 2012;124(3):243-6.

35. Otero S, Sulleiro E, Molina I, Espiau M, Suy A, Martin-Nalda A, et al. Congenital transmission of *Trypanosoma cruzi* in non-endemic areas: evaluation of a screening program in a tertiary care hospital in Barcelona, Spain. Am J Trop Med Hyg. 2012;87(5):832-6.

36. Rodari P, Angheben A, Gennati G, Trezzi L, Bargiggia G, Maino M, et al. Congenital Chagas disease in a non-endemic area: Results from a control programme in Bergamo province, Northern Italy. Travel Medicine and Infectious Disease. 2018;25:31-4.

37. Rubio M, Ebensperger I, Howard J, Knierim F, Naquira F. Search for Chagas' disease in 100 mothers of premature infants, with the finding of a case of congenital Chagas' disease. Boletin Chileno de Parasitologia. 1962;17:13-6.

38. Salas NA, Cot M, Schneider D, Mendoza B, Santalla JA, Postigo J, et al. Risk factors and consequences of congenital Chagas disease in Yacuiba, south Bolivia. Trop Med Int Health. 2007;12(12):1498-505.

39. Sasagawa E, Aiga H, Soriano EYC, Marroquín BLC, Ramírez MAH, de Aguilar AVG, et al. Mother-to-child transmission of chagas disease in El Salvador. The American Journal of Tropical Medicine and Hygiene. 2015;93(2):326-33.

40. Sosa-Estani S, Dri L, Touris C, Abalde S, Dell'arciprete A, Braunstein J. Vectorial and congenital transmission of *Trypanosoma cruzi* in Las Lomitas, Formosa. Medicina (B Aires). 2009;69(4):424-30.

41. Streiger M, Fabbro D, del Barco M, Beltramino R, Bovero N. Congenital Chagas disease in the city of Santa Fe. Diagnosis and treatment. Medicina (B Aires). 1995;55(2):125-32.

42. Tello P, Fernández P, Sandoval L, Ampuero G, Pizarro T, Schenone H. *Trypanosoma cruzi* infection in mother and child from the north section of Santiago, Chile. Boletin Chileno de Parasitología. 1982;37(1/2):23-4.

43. Torrico F, Alonso-Vega C, Suarez E, Rodriguez P, Torrico MC, Dramaix M, et al. Maternal *Trypanosoma cruzi* infection, pregnancy outcome, morbidity, and mortality of congenitally infected and non-infected newborns in Bolivia. Am J Trop Med Hyg. 2004;70(2):201-9.

44. Valenzuela M, Pinto M, Contreras M, Sandoval L, Silva M, Cerda G, et al. Enfermedad de Chagas en Chile. Sectores urbanos. VIII. Frecuencia de la infeccion por *Trypanosoma cruzi* en donantes de sangre en madres y recien nacidos de las ciudades de Rancagua, San Fernando y Santa Cruz. VI Region, 1983-1984. Bol Chil Parasitol. 1984:75-7.

45. Valperga SM, Castagnaro AE, Ovejero de Valperga GJ, Mirabella de Miotti MG, Arnau Enrico SC, Alonso BE, et al. Prevalencia de Chagas congénito: segundo estudio en Tucumán, Argentina. Cienc Méd(San Miguel de Tucumán). 1992:137-55.

46. Vicco MH, Rodeles L, Capovilla GS, Perrig M, Choque AGH, Marcipar I, et al. IgG autoantibodies induced by *T. cruzi* during pregnancy: Correlation with gravidity complications and early outcome assessment of the newborns. Maternal and Vhild Health Journal. 2016;20(10):2057-64.

47. Villablanca E, Osorio L, Salinas P. Chagas' disease in Chile. Urban sections. VII. Frequency of Chagasic infection in blood donors and mothers and newborns from the cities of San Felipe and Los Andes. V Region, 1983-1984. Bol Chil Parasitol. 1984;39(3-4):72-4.

48. Zaidenberg M, Segovia A. Congenital Chagas' disease in Salta, Argentina. Revista do Instituto de Medicina Tropical de São Paulo. 1993;35(1):35-43.
