# Supplementary material for: Estimation of the morbidity and mortality of congenital Chagas disease: A systematic review and meta-analysis
Source: PLoS Negl Trop Dis. 2022 Nov 7;16(11):e0010376. doi: 10.1371/journal.pntd.0010376 (PMC9671465; doi:10.1371/journal.pntd.0010376)
Supplement: S6 File — (DOCX) [file pntd.0010376.s006.docx]

**S6 File.** Sensitivity analyses results and assessment of heterogeneity

Results from sensitivity analyses are found in **Table 3**. Excluding studies with high risk of bias (n=13), the pooled proportion of infants with congenital Chagas disease (cCD) with clinical signs to all infants with cCD was 16.9% (95% CI 5.3%, 33.5%). Excluding mothers not diagnosed by WHO guidelines (n=28), the pooled proportion was 31.2% (95% CI 17.5%, 47.0%). Using the Miller back-transformation, the pooled proportion of infants with cCD with clinical signs to all infants with cCD was 23.2% (95% CI 12.1%, 35.8%) and the pooled proportion of mortality was 0.0% (95% CI = 0.0%,0.0%).

**Table 3. Sensitivity Analyses**

|  | **Pooled Proportion %** | 95% CI % | **I^2^ (%)** | 95% CI % | **Egger's Bias** | P-Value^*^ |
| --- | --- | --- | --- | --- | --- | --- |
| ***Primary Analyses (N=47)*** |  |  |  |  |  |  |
| Morbidity | 28.3 | 19.0,38.5 | 88.6 | 86.0,90.5 | 2.5 | <0.0001 |
| Mortality | 2.2 | 1.3,3.5 | 9.6 | 0.0,37.5 | 0.3 | 0.0084 |
| ***Sensitivity Analyses (N=47)*** |  |  |  |  |  |  |
| Low/Moderate Risk of Bias (n=13) | 16.9 | 5.3,33.5 | 91.4 | 89.2,93.0 | 1.9 | 0.0078 |
| WHO Recommended Maternal Diagnosis (n=28) | 31.2 | 17.5,47.0 | 91.2 | 87.3,93.5 | 2.7 | 0.0003 |
| ***Ad-Hoc Miller Transformation (N=47)*** |  |  |  |  |  |  |
| Morbidity | 23.2 | 12.1,35.8 | 88.0 | 86.0,90.5 | 2.5 | <0.0001 |
| Mortality | 0.0 | 0.0,0.0 | 7.8 | 0.0,36.0 | 0.3 | 0.0085 |

*^*^Egger's Bias plot statistical significance for asymmetry*

Our meta-analysis was performed using the Freeman-Tukey double arcsine method and applying Stuart-Ord inverse variance weights to transformed proportions. Using the Miller back-transformation, the estimated summary frequency decreased to 23.2% for morbidity and 0.0% for mortality. For morbidity, the summary frequency differs enough to conclude results are not robust and may be influenced by the variance transformation used. For mortality, however, results are robust. Differences in these values may be attributable to the fact there is high heterogeneity between studies for morbidity but may not have been a factor between studies for mortality.

In assessing the robustness of results to the decision to include studies with high risk of bias, studies presenting with only low or moderate risk of bias were analyzed. In so doing, a summary frequency of 16.9% was calculated from 13 included studies. This pooled proportion differs by nearly half when comparing to 28.3% and are thusly not robust, suggesting our results were influenced by risk of bias.

Regarding studies where maternal diagnosis of Chagas disease differed from WHO guidelines, a summary frequency of 31.2% was calculated from 28 included studies. This pooled proportion is similar to our primary findings and therefore results are robust and likely not influenced by method of maternal diagnosis for Chagas disease.

With regard to heterogeneity for subgroup analyses, for infants diagnosed using the gold standard method, the estimated pooled proportion of infants with cCD with clinical signs to all infants with cCD was 18.7% versus 32.5% for infants diagnosed by an alternative method. Fewer infants were found with clinical signs when diagnosed by the gold standard method, suggesting results may be influenced by infant diagnostic method. Additionally, the I^2^ values within each subgroup are considerably heterogenous (gold standard = 86.8% and alternative method = 78.9%), indicating substantial variability attributable to differences among diagnostic tests used for congenitally infected infants. Although overall highly heterogenous, it is interesting to note that both subgroups have a lower I^2^ value compared to the primary I^2^ value of 88.6%.

In the next subgroup analysis, the I^2^ statistic was calculated to measure the proportion of total variability attributable to heterogeneity in geographic area within each study. Based on geographic region, the pooled proportion of infants with cCD with clinical signs to all infants with cCD was 20.0% in studies based in Europe compared to 29.4% for studies based in Latin America. Furthermore, heterogeneity was unlikely a factor with studies based in Europe (I^2^ = 10.2%); however, studies based in the Latin American region were considerably heterogenous (I^2^ = 91.3%). These results draw mixed conclusions regarding variability attributable to differences between geographic locations.

For the subgroup analysis by individual clinical signs, the I^2^ values ranged from moderately to considerably heterogenous (jaundice = 59.9%, low birth weight = 60.7%, preterm birth = 61.2%, anemia = 64.1%, and hepatosplenomegaly = 85.8%), indicating some variability attributable to differences in type of clinical sign displayed in the infant.

The proportion of infants with cCD with clinical signs by year of study data collection was calculated in three timeframes: data collected prior to 2000, data collected from 2000 to 2010, and data collected past 2010. Considering heterogeneity, the I^2^ values ranged from not important to considerably heterogeneous (pre-2000 = 94.5%, 2000-2010 = 89.8%, post-2010 = 29.2%). This indicated that heterogeneity may not be important for those studies who collected data post-2010 but that heterogeneity was considerable for the studies whose data were collected prior to 2010.
