# Supplementary material for: Estimation of the morbidity and mortality of congenital Chagas disease: A systematic review and meta-analysis
Source: PLoS Negl Trop Dis. 2022 Nov 7;16(11):e0010376. doi: 10.1371/journal.pntd.0010376 (PMC9671465; doi:10.1371/journal.pntd.0010376)
Supplement: S1 Table — (DOCX) [file pntd.0010376.s008.docx]

**S1 Table.** Congenital cases morbidity characteristics

| **Article** | **Number Infected** | **Clinical Signs**  n(%) | **Morbidity**  n(%) | | | | | |
| --- | --- | --- | --- | --- | --- | --- | --- | --- |
|  |  |  | **Hepato-splenomegaly** | **PTB** | **LBW** | **Anemia** | **Jaundice** | **Other** |
| Apt 2013 [1] | 6 | 3(50.0) | 0(0.0) | 1(17.0)* | 0(0.0) | 0(0.0) | 0(0.0) | 1(17.0) - Transient hypotonia  2(33.0) - Respiratory symptomatology cyanosis and grunting  1(17.0) - Bronchopneumonia  1(17.0) - Respiratory distress |
| Azogue 1991 [2] | 78 | 21(27.0) | 35(45.0) | 0(0.0) | 0(0.0) | 0(0.0) | 11(14.0) | 4(5.0) - Edema  2(3.0) - Respiratory difficulties  4(5.0) - Other symptoms, undefined |
| Barona-Vilar 2012 [3] | 8 | 1(13.0) | 1(13.0) | 0(0.0) | 0(0.0) | 0(0.0) | 0(0.0) | Not specified |
| Barousse 1978 [4] | 1 | 1(100.0) | 1(100.0) | 1(100.0) | 1(100.0) | 0(0.0) | 0(0.0) | Not specified |
| Basile 2019^1^ [5] | 28 | 4(14.0) | 0(0.0) | 0(0.0) | 0(0.0) | 0(0.0) | 3(11.0) | Not specified |
| Bern 2009 [6] | 10 | 3(30.0) | 3(30.0) | 0(0.0) | 0(0.0) | 0(0.0) | 0(0.0) | Not specified |
| Bittencourt 1985 [7] | 3 | 2(67.0) | 1(33.0) | 2(67.0) | 2(67.0) | 1(33.0) | 1(33.0) | 1(33.0) - Hyporeflexia  1(33.0) - Dyspnea  1(33.0) - Association with perinatal hemolytic disease |
| Buekens 2018 [8] | 11 | 4(36.0) | 1(9.0) | 1(9.0) | 0(0.0) | 0(0.0) | 1(9.0) | 1(9.0) - Sepsis  1(9.0) - Apgar <7 at 1-minute  1(9.0) - NICU admission  3(27.0) – PROM |
| Cardoso 2012 [9] | 15 | 1(7.0) | 0(0.0) | 1(7.0) | 0(0.0) | 0(0.0) | 0(0.0) | Not specified |
| Castillo 1984 [10] | 31 | 2(6.0) | 0(0.0) | 0(0.0) | 2(6.0) | 0(0.0) | 0(0.0) | Not specified |
| Flores-Chavez 2011 [11] | 4 | 1(25.0) | 0(0.0) | 0(0.0) | 0(0.0) | 0(0.0) | 0(0.0) | 1(25.0) - Downs syndrome  1(25.0) - Congenital cardiopathy |
| Francisco-González 2019 [12] | 3 | 1(33.0) | 0(0.0) | 0(0.0) | 0(0.0) | 1(33.0) | 0(0.0) | 1(33.0) - Hydrops fetalis  1(33.0) - Ascites  1(33.0) - Hemodynamic instability  1(33.0) - NICU admission |
| Freilij 1995 [13] | 71 | 25(35.0) | 13(18.0) | 0(0.0) | 0(0.0) | 1(1.0) | 0(0.0) | 5(7.0) - Sepsis  3(4.0) - Hepatitis  1(1.0) - Edema  3(4.0) - HIV co-infection  1(1.0) - Respiratory distress |
| Giménez 2010 [14] | 3 | 1(33.0) | 0(0.0) | 0(0.0) | 0(0.0) | 0(0.0) | 0(0.0) | 1(33.0) - Dilated cardiomyopathy  1(33.0) - Neuroblastoma |
| Martínez de Tejada 2009 [15] | 2 | 1(50.0) | 1(50.0) | 0(0.0) | 0(0.0) | 0(0.0) | 0(0.0) | Not specified |
| Mayer 2010 [16] | 18 | 9(50.0) | 7(39.0) | 0(0.0) | 0(0.0) | 0(0.0) | 0(0.0) | 4(22.0) - Cardiomyopathy |
| Messenger 2017^2^ [17] | 38 | 11(29.0) | 0(0.0) | 6(19.0) | 7(22.0) | 0(0.0) | 0(0.0) | 1 - Apgar <7 at 1-minute  5/32 - NICU admission  4(13.0) - PROM |
| Munoz 1982 [18] | 2 | 2(100.0) | 0(0.0) | 1(50.0) | 1(50.0) | 1(50.0) | 0(0.0) | 1(50.0) - Central nervous system problems  1(50.0) - Possible overload of left ventricle |
| Nisida 1999 [19] | 4 | 4(100.0) | 1(25.0) | 1(25.0) | 1(25.0) | 2(50.0) | 0(0.0) | 1(25.0) - Femur metaphysis  1(25.0) - Seizure  1(25.0) - Stillbirth >20 weeks  1(25.0) - Spontaneous abortion <20 weeks |
| Otero 2012 [20] | 1 | 1(100.0) | 1(100.0) | 1(100.0) | 1(100.0) | 0(0.0) | 0(0.0) | 1(100.0) - Cholestasis  1(100.0) - Cytolysis |
| Rodari 2018 [21] | 1 | 1(100.0) | 0(0.0) | 1(100.0) | 0(0.0) | 1(100.0) | 0(0.0) | Not specified |
| Rubio 1962 [22] | 1 | 1(100.0) | 1(100.0) | 0(0.0) | 0(0.0) | 0(0.0) | 0(0.0) | 1(100.0) - Bronchopneumonia  1(100.0) - Hepatic steatosis |
| Salas 2007 [23] | 58 | 15(31.0) | 0(0.0) | 0(0.0) | 0(0.0) | 13(22.0) | 0(0.0) | Not specified |
| Sosa-Estani 2009 [24] | 8 | 2(25.0) | 0(0.0) | 0(0.0) | 1(13.0) | 0(0.0) | 0(0.0) | 1(13.0) - Dehydration  1(13.0) - Sepsis  1(13.0) - Respiratory distress  1(13.0) - Gastroenteritis  1(13.0) - Scarce clear meconial liquid |
| Streiger 1995 [25] | 9 | 6(67.0) | 3(33.0) | 5(56.0) | 6(67.0) | 0(0.0) | 1(11.0) | 1(11.0) - Respiratory difficulty  1(11.0) - Malnourished  1(11.0) - Pneumopathy (lung disease) |
| Torrico 2004 [26] | 71 | 36(51.0) | 21(30.0) | 3(4.0) | 4(6.0) | 0(0.0) | 0(0.0) | 9(13.0) - Cardiomegaly  2(3.0) - Apgar score 0 at 5-minute  8(11.0) - Neurological signs  6(8.0) - Anasarca  6(8.0) - Petechia |
| Valenzuela 1984 [27] | 11 | 4(36.0) | 0(0.0) | 0(0.0) | 3(27.0)* | 0(0.0) | 0(0.0) | Not specified |
| Valperga 1992 [28] | 4 | 3(75.0) | 1(25.0) | 1(25.0) | 1(25.0) | 0(0.0) | 2(50.0) | 1(25.0) - Maxillofacial malformation  2(50.0) - SGA |
| Villablanca 1984 [29] | 61 | 25(41.0) | 0(0.0) | 0(0.0) | 25(41.0) | 0(0.0) | 0(0.0) | Not specified |
| Zaidenberg 1993 [30] | 12 | 12(100.0) | 12(100.0) | 5(42.0) | 0(0.0) | 10(83.0) | 10(83.0) | 2(17.0) - SGA |

*PTB*, preterm birth; *MPTB*, moderately preterm birth; *VPTB*, very preterm birth; *EPTB*, extremely preterm birth; *LBW*, low birthweight; *NICU*, neonatal intensive care unit; *PROM*, premature rupture of membranes; *SGA*, small for gestational age.

A value of 0 was inferred from missing data, as most studies only explicitly outlined symptoms that were present.

PTB counts any report of PTB <37 weeks), MPTB(32-36 weeks), VPTB(28-31 weeks), EPTB(<28 weeks).

LBW counts any report of LBW (<2500g), VLBW (<1500g), ELBW(<1000g).

Hepatosplenomegaly counts any report of hepatomegaly, splenomegaly, and hepatosplenomegaly.

^1^Unable to determine total number of infants with hepatosplenomegaly.

^2^Study reported 38 infected cases and symptoms were reported out of 32 infected cases.

*Undefined LBW and PTB.

**References**

1. Apt W, Zulantay I, Arnello M, Oddó D, González S, Rodríguez J, et al. Congenital infection by *Trypanosoma cruzi* in an endemic area of Chile: a multidisciplinary study. Transactions of the Royal Society of Tropical Medicine and Hygiene. 2013;107(2):98-104.

2. Azogue E, Darras C. Prospective study of Chagas disease in newborn children with placental infection caused by Trypanosoma cruzi (Santa Cruz-Bolivia). Rev Soc Bras Med Trop [Internet]. 1991 [cited 2022 Oct 24];24(2):105–9. Available from: https://pubmed.ncbi.nlm.nih.gov/1841425/

3. Barona-Vilar C, Gimenez-Marti MJ, Fraile T, Gonzalez-Steinbauer C, Parada C, Gil-Brusola A, et al. Prevalence of *Trypanosoma cruzi* infection in pregnant Latin American women and congenital transmission rate in a non-endemic area: the experience of the Valencian Health Programme (Spain). Epidemiol Infect. 2012;140(10):1896-903.

4. Barousse A, Eposto M, Mandel S, Martínez F. Congenital Chagas' disease in a non-endemic area. Medicina. 1978;38(6 Pt 1):611-5.

5. Basile L, Ciruela P, Requena-Méndez A, Vidal MJ, Dopico E, Martín-Nalda A, et al. Epidemiology of congenital Chagas disease 6 years after implementation of a public health surveillance system, Catalonia, 2010 to 2015. Eurosurveillance. 2019;24(26):1900011.

6. Bern C, Verastegui M, Gilman RH, Lafuente C, Galdos-Cardenas G, Calderon M, et al. Congenital *Trypanosoma cruzi* transmission in Santa Cruz, Bolivia. Clin Infect Dis. 2009;49(11):1667-74.

7. Bittencourt ACL, Mota E, Ribeiro Filho R, Fernandes LG, Almeida PRCd, Sherlock ÍRdA, et al. Incidence of congenital Chagas' disease in Bahia, Brazil. 1985.

8. Buekens P, Cafferata ML, Alger J, Althabe F, Belizán JM, Bustamante N, et al. Congenital transmission of *Trypanosoma cruzi* in Argentina, Honduras, and Mexico: an observational prospective study. The American Journal of Tropical Medicine and Hygiene. 2018;98(2):478-85.

9. Cardoso EJ, Valdéz GC, Campos AC, de la Luz Sanchez R, Mendoza CR, Hernández AP, et al. Maternal fetal transmission of *Trypanosoma cruzi*: a problem of public health little studied in Mexico. Experimental Parasitology. 2012;131(4):425-32.

10. Castillo S, Mardones C, Hormazábal G, Cubillos R, Barahona N, Zepeda S, et al. Chagas' disease in Chile. Urban sectors. VI. Frequency of Chagas' infection in blood donors and in mothers and newborn infants of the cities of Antofagasta and Calama. II Region (1983-1984). Boletin Chileno de Parasitologia. 1984;39(1-2):28-32.

11. Flores-Chavez MD, Merino FJ, García-Bujalance S, Martin-Rabadan P, Merino P, Garcia-Bermejo I, et al. Surveillance of Chagas disease in pregnant women in Madrid, Spain, from 2008 to 2010. Euro Surveill. 2011;16(38).

12. Francisco-González L, Gastañaga-Holguera T, Montero BJ, Pérez ZD, Ramos MI, Amador PM, et al. Seroprevalence and vertical transmission of Chagas disease in a cohort of Latin-American pregnant women in a tertiary hospital in Madrid. Anales de Pediatría (English Edition). 2018;88(3):122-6.

13. Freilij H, Altcheh J. Congenital Chagas' disease: diagnostic and clinical aspects. Clinical Infectious Diseases. 1995;21(3):551-5.

14. Giménez MJ, Gómez-Ruiz MD, Calabuig A, Perez-Tamarit A, Otero MC, Fernández-Silveira J, et al., editors. Congenital transmission of Chagas’ disease in Latin American immigrants in a health department of Valencia, Spain. European Congress of Clinical Microbiology and Infectious Diseases; 2010; Vienna, AT: Clinical Microbiology and Infection.

15. Martínez de Tejada B, Jackson Y, Paccolat C, Irion O. Congenital Chagas disease in Geneva: diagnostic and clinical aspects. Revue Medicale Suisse. 2009;5(222):2091-2, 4.

16. Mayer JP, Biancardi M, Altcheh J, Freilij H, Weinke T, Liesenfeld O. Congenital infections with *Trypanosoma cruzi* or Toxoplasma gondii are associated with decreased serum concentrations of interferon-c and interleukin-18 but increased concentrations of interleukin-10. Annals of Tropical Medicine & Parasitology. 2010;104(6):485-92.

17. Messenger LA, Gilman RH, Verastegui M, Galdos-Cardenas G, Sanchez G, Valencia E, et al. Toward improving early diagnosis of congenital Chagas disease in an endemic setting. Clin Infect Dis. 2017;65(2):268-75.

18. Munoz P, Lorca M, Thiermann E, Astorga B, Arias A, Pino S. Transmisión congénita del *Trypanosoma cruz*i: Investigación en la maternidad del Hospital San Juan de Dios, de Santiago. Revista Chilena de Pediatría. 1982;53(1-6):22-7.

19. Nisida IVV, Amato Neto V, Braz LMA, Duarte MIS, Umezawa ES. A survey of congenital Chagas’ disease, carried out at three Health Institutions in São Paulo City, Brazil. Revista do Instituto de Medicina Tropical de São Paulo. 1999;41(5):305-11.

20. Otero S, Sulleiro E, Molina I, Espiau M, Suy A, Martin-Nalda A, et al. Congenital transmission of *Trypanosoma cruzi* in non-endemic areas: evaluation of a screening program in a tertiary care hospital in Barcelona, Spain. Am J Trop Med Hyg. 2012;87(5):832-6.

21. Rodari P, Angheben A, Gennati G, Trezzi L, Bargiggia G, Maino M, et al. Congenital Chagas disease in a non-endemic area: Results from a control programme in Bergamo province, Northern Italy. Travel Medicine and Infectious Disease. 2018;25:31-4.

22. Rubio M, Ebensperger I, Howard J, Knierim F, Naquira F. Search for Chagas' disease in 100 mothers of premature infants, with the finding of a case of congenital Chagas' disease. Boletin Chileno de Parasitologia. 1962;17:13-6.

23. Salas NA, Cot M, Schneider D, Mendoza B, Santalla JA, Postigo J, et al. Risk factors and consequences of congenital Chagas disease in Yacuiba, south Bolivia. Trop Med Int Health. 2007;12(12):1498-505.

24. Sosa-Estani S, Dri L, Touris C, Abalde S, Dell'arciprete A, Braunstein J. Vectorial and congenital transmission of *Trypanosoma cruzi* in Las Lomitas, Formosa. Medicina (B Aires). 2009;69(4):424-30.

25. Streiger M, Fabbro D, del Barco M, Beltramino R, Bovero N. Congenital Chagas disease in the city of Santa Fe. Diagnosis and treatment. Medicina (B Aires). 1995;55(2):125-32.

26. Torrico F, Alonso-Vega C, Suarez E, Rodriguez P, Torrico MC, Dramaix M, et al. Maternal *Trypanosoma cruzi* infection, pregnancy outcome, morbidity, and mortality of congenitally infected and non-infected newborns in Bolivia. Am J Trop Med Hyg. 2004;70(2):201-9.

27. Valenzuela M, Pinto M, Contreras M, Sandoval L, Silva M, Cerda G, et al. Enfermedad de Chagas en Chile. Sectores urbanos. VIII. Frecuencia de la infeccion por *Trypanosoma cruzi* en donantes de sangre en madres y recien nacidos de las ciudades de Rancagua, San Fernando y Santa Cruz. VI Region, 1983-1984. Bol Chil Parasitol. 1984:75-7.

28. Valperga SM, Castagnaro AE, Ovejero de Valperga GJ, Mirabella de Miotti MG, Arnau Enrico SC, Alonso BE, et al. Prevalencia de Chagas congénito: segundo estudio en Tucumán, Argentina. Cienc Méd(San Miguel de Tucumán). 1992:137-55.

29. Villablanca E, Osorio L, Salinas P. Chagas' disease in Chile. Urban sections. VII. Frequency of Chagasic infection in blood donors and mothers and newborns from the cities of San Felipe and Los Andes. V Region, 1983-1984. Bol Chil Parasitol. 1984;39(3-4):72-4.

30. Zaidenberg M, Segovia A. Congenital Chagas' disease in Salta, Argentina. Revista do Instituto de Medicina Tropical de São Paulo. 1993;35(1):35-43.
