# Supplementary material for: Estimation of the morbidity and mortality of congenital Chagas disease: A systematic review and meta-analysis
Source: PLoS Negl Trop Dis. 2022 Nov 7;16(11):e0010376. doi: 10.1371/journal.pntd.0010376 (PMC9671465; doi:10.1371/journal.pntd.0010376)
Supplement: S2 Table — (DOCX) [file pntd.0010376.s009.docx]

**S2 Table.** Congenital cases mortality characteristics

| Article | **#** | **Mortality** | |
| --- | --- | --- | --- |
|  |  | **Time** | **Cause** |
| Bittencourt 1985 [1] | 1 | Birth | Stillbirth |
| Flores-Chavez 2011 [2] | 1 | 9 months | Born with Down’s syndrome and congenital cardiopathy. Suddenly died after recovering from congenital *Trypanosoma cruzi* infection |
| Freilij 1995 [3] | 2 | 8 months | Respiratory distress |
|  |  | 14 months | Severe neurological damage |
| Nisida 1999 [4] | 2 | Birth | Chagas disease |
|  |  | 30 Days | Chagas disease |
| Salas 2007 [5] | 2 | Date undetermined | Not specified |
| Sosa-Estani 2009 [6] | 2 | 4 months | Gastroenteritis and dehydration |
|  |  | 5 months | Pneumonia |
| Streiger 1995 [7] | 1 | 2 months | Secondary septicemia and pneumococcal meningitis |
| Torrico 2004 [8] | 4 | 24-48 hours | Chagas disease |

**References**

1. Bittencourt ACL, Mota E, Ribeiro Filho R, Fernandes LG, Almeida PRCd, Sherlock ÍRdA, et al. Incidence of congenital Chagas' disease in Bahia, Brazil. 1985.

2. Flores-Chavez MD, Merino FJ, García-Bujalance S, Martin-Rabadan P, Merino P, Garcia-Bermejo I, et al. Surveillance of Chagas disease in pregnant women in Madrid, Spain, from 2008 to 2010. Euro Surveill. 2011;16(38).

3. Freilij H, Altcheh J. Congenital Chagas' disease: diagnostic and clinical aspects. Clinical Infectious Diseases. 1995;21(3):551-5.

4. Nisida IVV, Amato Neto V, Braz LMA, Duarte MIS, Umezawa ES. A survey of congenital Chagas’ disease, carried out at three Health Institutions in São Paulo City, Brazil. Revista do Instituto de Medicina Tropical de São Paulo. 1999;41(5):305-11.

5. Salas NA, Cot M, Schneider D, Mendoza B, Santalla JA, Postigo J, et al. Risk factors and consequences of congenital Chagas disease in Yacuiba, south Bolivia. Trop Med Int Health. 2007;12(12):1498-505.

6. Sosa-Estani S, Dri L, Touris C, Abalde S, Dell'arciprete A, Braunstein J. Vectorial and congenital transmission of *Trypanosoma cruzi* in Las Lomitas, Formosa. Medicina (B Aires). 2009;69(4):424-30.

7. Streiger M, Fabbro D, del Barco M, Beltramino R, Bovero N. Congenital Chagas disease in the city of Santa Fe. Diagnosis and treatment. Medicina (B Aires). 1995;55(2):125-32.

8. Torrico F, Alonso-Vega C, Suarez E, Rodriguez P, Torrico MC, Dramaix M, et al. Maternal *Trypanosoma cruzi* infection, pregnancy outcome, morbidity, and mortality of congenitally infected and non-infected newborns in Bolivia. Am J Trop Med Hyg. 2004;70(2):201-9.
