# Supplementary material for: Estimation of the morbidity and mortality of congenital Chagas disease: A systematic review and meta-analysis
Source: PLoS Negl Trop Dis. 2022 Nov 7;16(11):e0010376. doi: 10.1371/journal.pntd.0010376 (PMC9671465; doi:10.1371/journal.pntd.0010376)
Supplement: Attachment — Submitted filename: Response to reviewers_04AUG2022.docx [file pntd.0010376.s010.docx]

| Comment | Response |
| --- | --- |
| Methods | |
| Reviewer #1: This is a paper reporting the results of a systematic review and metanalysis over a relevant neglected tropical disease, as it is Chagas disease, and over an even more relevant topic such as congenital Chagas disease. The important contribution that is reported in this paper is the proportion of infants diagnosed with congenital Chagas disease at birth presenting with clinical symptoms, which was unknown prior this study. | Thank you for your comment. |
| Reviewer #1: Here some recommendations:  Page 3, Line 46: Take note that food can be contaminated by different ways (triatomine feces, crushed triatomines, anal gland secretions from infected marsupials). A more accurate statement could be: ......via oral consumption of contaminated food or beverage, .... | Thank you for your feedback – we have modified this line to the recommended change. |
| Reviewer #1: Here are some recommendations: There are several sections in which the following statement is used: cCD infected infants. The correct way to refer to this information would be infants with cCD or congenitally T. cruzi infected children. Please, review this information in Lines 54, 108, 220 (two times), 268-269 (two times), 340, 348 | Thank you for your feedback. We have modified the referenced lines and the remainder of the paper to the correct terminology. |
| Reviewer #1: Here are some recommendations: Page 25, Line 384: include the institution and country information per each person being acknowledged. | Thank you for noting this. We have added the institution and country information for each person acknowledged. |
| Reviewer #3: This is an interesting systematic review and meta-analysis aiming to identify studies that collected information on infants with congenital Chagas disease. The objective of this systematic review is to determine the morbidity and mortality of cCD and it is clearly stated. The authors have summarized and analyzed 47 eligible studies; the information about the populations and sample sizes is in general well described. However it would be interesting to know how many studies have provided information regarding the phylogenetic lineage or discrete typing unit of the Trypanosoma cruzi populations, and if any association could be found between certain discrete typing units and the occurrence of signs and symptoms of disease. This should be in relation to the geographical distribution of cCD cases with morbidity signs and symptoms among endemic countries (In fact, a high proportion of cCD infants born in European countries are born to mothers infected in Bolivia, where certain parasite genotypes prevail). To analyse the frequency of signs and symptoms according to regions where T.cruzi I, T.cruzi II or T.cruzi V and or T.cruzi VI circulate would be a plus in the analysis. | Thank you for this suggestion. We considered a post-hoc analysis of included studies to analyze the proportion of infants with cCD with clinical signs by discrete typing unit of T. cruzi. Unfortunately, we were unable to proceed with analysis as only one eligible study included any data on this topic. We have noted this lack of information as a limitation of our study. |
| Reviewer #4: The objective of the study are clearly articulated with testable hypothesis stated. The study design which is a meta-analysis is appropriate and to address the stated objectives. The statistics of the research are clear, understandable and verifiable. There is no concerns about ethical or regulatory requirements. | Thank you for you feedback. |
| Results | |
| Reviewer #1: The authors performed pooled analysis using several approaches to measure the different outcomes (frequency, proportion) as well as assessment of heterogeneity between studies and sensitivity analysis of results. | Thank you for your comment. |
| Reviewer #2: The results are clearly presented and the Tables and Figures are adequate. | We appreciate your feedback. |
| Reviewer #4: The results of the study are presented clearly and completely. Tables and figures are of sufficient quality for clarity. The presented analysis matches the analysis plan. | Thank you for your comment. |
| Conclusions | |
| Reviewer #1: The authors acknowledge the limitations of their approach. The conclusions are based on the evidence presented. | We appreciate your feedback. |
| Reviewer #3: The strengths and weaknesses of the study are clearly expressed in the Discussion. It would be desirable that, al least, any comment about the parasite diversity in relation of cCD transmission and morbidity be taken into account in the discussion. | Thank you for your feedback. We have taken note of the lack of information on infants with cCD that present with clinical signs as a limitation of our study. |
| Reviewer #1: Minor observations: Page 3, Line 28: it is necessary to add the abbreviation after congenital Chagas disease: …information on infants with congenital Chagas disease (cCD), we summarized… | Thank you for your comment. We have added the abbreviation where indicated. |
| Reviewer #1: Do not start a sentence with a number or an abbreviation. If you need to start a sentence with a number or an abbreviation such as a scientific name, present the number in letters and write the complete name. Please review the whole document. Examples of sentences to be reviewed include Lines 43, 49, 188, 194-196, 203, | Thank you noting this – we have corrected this throughout our manuscript. |
| Reviewer #1: Page 5. Line 90: Please review the correct wording: In 2010, it was estimated that between… | Thank you for your comment – we have reviewed and corrected the wording where indicated. |
| Reviewer #1: Page 6, Line 106: Include the PROSPERO registry number in the text. | Thank you for your feedback. We have added the PROSPERO registry number in the text. |
| Reviewer #1: Page 6, Line 115: To be accurate, include that it refers to congenital infection T. cruzi congenitally infected infant | Thank you for your comment – we have added this where indicated. |
| Reviewer #1: Page 21, Line 263: the abbreviation LBW has not been used before in the manuscript. Better to write low birth weight | Thank you for your comment. We have added in the full wording followed by its abbreviation for clarity. |
| Reviewer #1: Page 22, Line 299: review wording: Chagas disease Page 22, Line 302: the word historically is used two times in the same sentence. | Thank you for your feedback. We have corrected this error. |
| Reviewer #1: Page 24, Line 354: review wording: the comparison to a non-infected control group | Thank you for your comment. We have reviewed and corrected the wording. |
| Reviewer #1: S1 File – Complete the name of the abbreviation ICU | Thank you for your feedback. We have completed the full name of intensive care unit and placed its abbreviation after. |
| Reviewer #1: S1 Table – Complete the name of abbreviations such as NICU, PROM; PTB, MPTB, VPTB, EPTB, LBW, VLBW, ELBW Review the spelling of the words: anasarca, maxillofacial, pneumopathy, bronchopneumonia, metaphysis | Thank you for noting this. We have corrected the spelling of indicated words and added in the full name for all included abbreviations prior to their reference in this manuscript. |
| Reviewer #1: S2 File – Confirm the search term in LILACS: Tiypanosom$ | Thank you for your feedback. This has been corrected. |
| Reviewer #1: S5 File – Complete the name of abbreviation: Participants SES | Thank you for your feedback. We have added the full name prior to using this abbreviation. |
| Reviewer #3: Minor revision: Line 90 : Rational . It was estimated instead of its estimated. | Thank you for your comment. We have made the suggested correction. |
| Reviewer #3: Line 142.. Please clarify this part of the sentence : independently assessed included studies’ risk of bias of the included studies | Thank you for your feedback. We have clarified here that we had two reviewers independently assess every included studies’ risk of bias to the results of this study. |
| Summary and General Comments | |
| Reviewer #1: This is a paper reporting the results of a systematic review and metanalysis over a relevant neglected tropical disease, as it is Chagas disease, and over an even more relevant topic such as congenital Chagas disease. The important contribution that is reported in this paper is the proportion of infants diagnosed with congenital Chagas disease at birth presenting with clinical symptoms, which was unknown prior this study. The authors performed pooled analysis using several approaches to measure the different outcomes (frequency, proportion) as well as assessment of heterogeneity between studies and sensitivity analysis of results. The authors also acknowledge the limitations of their approach. The conclusions are based on the evidence presented. | We appreciate your feedback. |
| Reviewer #2: This manuscript describes the results of a metaanalysis of morbidity and mortality associated with congenitally acquired Chagas disease. The authors have gone to great effort to search the published scientific literature and extract analyzable data from a small subset of the studies they found. As they clearly acknowledge, there are concerns for sources of bias in the analysis but overall their conclusions about manifestations and deaths are informative. They attempted to examine the influence of several factors that might cause heterogeneity but failed to include one potential source, year of publication (better would be year(s) during which the study was performed). Given the wide range of time covered, it is very likely that level of health care, prevalence of comorbid conditions, and other factors may have changed in a given geographic area which would potentially influence outcomes. Especially in the discussion section, the differences between public health disease control programs and research studies is not clear. The lack of better data on clinical manifestations and outcomes of congenital Chagas disease is due to both under resourced country programs and paucity of robust research studies. The advocacy targets and messages for the two are very different. | Thank you for your feedback. Per your suggestion, we have conducted a post-hoc analysis of the proportion of infants with cCD that present with clinical signs by year(s) of study data collection. The results of this analysis have been included and we address the various time influences mentioned that could have affected these results.  In our discussion, we have taken into consideration your comments and added in clarification on when we refer to public health disease control programs and research studies. Per your suggestion, we have made an effort to distinguish the advocacy targets for improving data via strengthening country programs and more robust research studies. |
| Reviewer #2: Pg. 4 lines 53-63. Although the term is often used in disease descriptions for the general public, in most medical literature symptoms are defined as subjective, as perceived by the patient, which would not pertain to newborn infants. The term can be used to describe a condition that is an indication of an underlying process. This does not seem to be what is described here. | Thank you for this advice. We have changed this throughout our manuscript to say “with clinical signs” rather than signs and symptoms. |
| Reviewer #2: Pg. 4 lines 69-71. Antibody development may be subsequent to the parasitemia of acute infection. Acute infection is diagnosed by detection of the parasite, either morphologically or by molecular testing (PCR) and patients will have positive results on PCR before they have detectable antibody levels. Diagnosis is clearly described in PAHO 2019 Guidelines for the diagnosis and treatment of Chagas disease and Forsyth et al. J Infect Dis 2022 Recommendations for screening and diagnosis of Chagas disease in the United States. | Thank you for this feedback. We have reviewed the referenced guidelines and found that PAHO has cited the gold standard for diagnosis of cCD requires, at birth, parasitological examination using microhematocrit or microStrout testing methods, and if negative repeated parasitological examination one month later, and starting at 8-10 months of age two serological tests. Per their 2019 guidelines, molecular methods of diagnosis using PCR are not considered part of the gold standard diagnosis. The cited 2022 guidelines are specific to the United States, and given that the majority of studies we have included were conducted outside of this region. In addition, our analysis was completed prior to the publication of Forsyth et al., and the studies included in our analysis are not in the United States, we felt that these recommendations were not pertinent to our study and thus this was not included. Thus, we have decided to follow the PAHO 2019 guidelines for this study. |
| Reviewer #2: Pg. 4 lines 71-73. The currently available rapid diagnostic tests are for antibody detection, please see comment above regarding diagnosis of acute infection. Depending on the setting, rapid tests may be used for as the first test (see PAHO Guidelines). | Thank you for your feedback. We have addressed this comment above. |
| Reviewer #2: Pg. 5 line 79. This is either the Strout method or microStrout method, capitalization is needed. | Thank you for your comment. We have corrected this in our manuscript. |
| Reviewer #2: Pg. 5 lines 81-83. I could not find the evidence to support this statement regarding molecular methods for diagnosis of congenital Chagas disease in the cited reference. | Thank you for this feedback, we have adjusted the language to better reflect the evidence in the cited reference. |
| Reviewer #2: Pg. 5 line 90. Do you mean it is estimated? Since the cited evidence was from 12 years ago, it would be more accurate to say it was estimated. | Thank you, we have made the suggested correction. |
| Reviewer #2: Pg. 6 line 109. The term is randomized controlled trials, no hyphen. | Thank you for your feedback, this has been corrected. |
| Reviewer #2: Pg. 6 line 113-114. A study would be designed to collect original data or not and that design would be a criterion for inclusion; this sentence seems out of place in outcomes. Weren’t “articles” the published study results/outcomes? | Thank you for your feedback. We have made the suggested change in language to articles here. |
| Reviewer #2: Pg. 7 line 125. Do you mean article title? In most published scientific literature, the study that generated the reported results may or may not have had a specific title that would be provided in the written report. At lines 128-129, were the studies duplicated or were the results from the same study published in “duplicate” articles? In the S3 document, articles were duplicates. In some cases, an individual study has had results published more than once, perhaps that was what you meant? Either S3 or the text at lines 128-129 need to be revised to agree. | Thank you, we have corrected the suggested changes. We have further clarified that articles reporting on the same study population at different time periods (ie., duplicate articles of the same study population), the one with the largest sample size was included. Duplicate articles in S3 included these as well as articles that were published more than once. Duplicates of articles that reported on the same study population within the same timeframe were removed to make sure the same study population was only considered once. |
| Reviewer #2: Pg. 7 line 143 I think a copy and paste error, included studies appears twice. | Thank you, we have corrected this error. |
| Reviewer #2: Pg. 8 line 149. The topics were narratively summarized, not was. | Thank you, we have made the suggested correction. |
| Reviewer #2: Pg. 8 lines 188-189. Please consider revising to avoid confusion over the term Caribbean region; many readers will interpret that to include the Caribbean islands. | Thank you for this comment. We have revised the entire manuscript to ensure consistency in this language and removed the term Caribbean region to avoid confusion. |
| Reviewer #2: Pg. 16 line 220. It would be more accurate to compare infants with detected clinical manifestations to those without, or infants with signs to those without. | Thank you, we have corrected this to reflect that we assessed the proportion of infants with cCD with clinical signs in all studies. |
| Reviewer #2: Pg. 16 Table 2. Please provide a title that orients the reader and better descriptions for column one row titles, even if only with footnotes. The reader has to go back to the methods section to understand what is meant by primary and subgroup analysis and how to interpret “Infant gold” or “Infant other”. | Thank you, we have adjusted our tables to ensure ease of interpretation for the reader. |
| Reviewer #2: Pgs. 17-20 Figs. 1-4. Please provide descriptive titles and legends for all figures to orient readers. | Thank you, we have adjusted our tables to ensure ease of interpretation for the reader. |
| Reviewer #2: Pg. 20 line 257. Please revise, Caribbean is not accurate. | Thank you, we have changed this phrasing to remove Caribbean. |
| Reviewer #2: Pg. 20-21 lines 259-264. Here symptom is used to categorize what was defined as sign on pg. 4. Please revise for consistency and to reflect that infants are not reporting symptoms, but clinical signs may be recognized or diagnosed by the health care provider or caregiver. | Thank you, we have corrected throughout the manuscript that we are referring to infants with cCD with or without clinical signs. |
| Reviewer #2: Pg. 22 lines 299-309. This paragraph should be revised or deleted.The WHO designation was intended to draw attention to the neglect, not cause it; Consider revising, perhaps something like this: Chagas disease primarily affects impoverished populations and few resources have been dedicated to addressing the disease, despite the World Health Organization (WHO) defining Chagas disease a neglected tropical disease. | Thank you for this feedback. We have edited this paragraph to reflect your suggested change and ensure clear understanding of this point. |
| Reviewer #2: There is no second mentioned so no need to say “first” (line 299). | Thank you, we have corrected this error. |
| Reviewer #2: The word historically appears twice in the same sentence, please revise (line 302). | Thank you, we have corrected this error. |
| Reviewer #2: lines 303-304. Not the Caribbean, please correct throughout this manuscript. Why is 2017 in parentheses here? Most studies, poor quality or not, and their published results are not produced as part of ongoing disease control programs. However, consistently funded and supported effective public health programs that cover the jurisdictions needs, as the cited reference advocates, can generate reliable data to monitor disease trends. | Thank you, we have removed Caribbean from the phrasing and corrected the cited error. We have shifted our discussion here to reflect that we are referring to the need to increase the quality of the body of cCD literature. |
| Reviewer #2: lines 304-306. Consider using the original references that were cited in the AHA position statement overview of Chagas disease. | Thank you for your comment. In our revision of this paragraph, this sentence and reference has been removed. |
| Reviewer #2: lines 308-309. Here the underfunding of disease control and prevention programs are again blamed for a lack of research and studies of Chagas disease. Please revise. | Thank you, we have revised this to reflect that we are referring to the effect that the historical lack of emphasis on prevention of cCD has had on the quality of research conducted on its prevention prior to major regional disease control programs in Latin America. We agree these are two separate issues and have revised our interpretation to emphasize a need for further resources needed, and separately, the need for robust research studies. |
| Reviewer #3: The meta-analysis allowed estimation of 28.3% of infants with symptomatic congenital Chagas disease and 2.2% with fatal outcome. Such integrative amount of data about the clinical compromise observed in Congenital Chagas disease infants has not been reported before, so this manuscript can be of true value to the health community working on neglected tropical diseases. | Thank you for your feedback. |
