# Supplementary material for: Estimation of the morbidity and mortality of congenital Chagas disease: A systematic review and meta-analysis
Source: PLoS Negl Trop Dis. 2022 Nov 7;16(11):e0010376. doi: 10.1371/journal.pntd.0010376 (PMC9671465; doi:10.1371/journal.pntd.0010376)
Supplement: Attachment — Submitted filename: Response to reviewers_24SEP2022.docx [file pntd.0010376.s011.docx]

| **Comment** | **Response** |
| --- | --- |
| **Methods** | |
| Reviewer #2: No comments. | Thank you. |
| Reviewer #4: The objectives of the study are clearly stated with a testable hypothesis. The study design was designed in accordance with the stated objectives. The population included in the study was clearly defined and suitable for the hypotase tested. The sample size included in the study is sufficient to provide sufficient test power to address the hypothesis being tested. The statistical analyzes used are appropriate and accurate. There is no ethical concern. | Thank you for your comment! |
| **Results** | |
| Reviewer #2: No comments. | Thank you. |
| Reviewer #4: Analyzes were prepared in accordance with the analysis methods. The results are clear and straightforward. Tables are adequate and of sufficient quality. | Thank you for your comment! |
| **Conclusions** | |
| Reviewer #2: Thank you for including time (year of data collection/publication) in your analyses. However, the interpretation of the differences you found is concerning. Lines 403-404. The differences in studies’ results over time are a limitation of this metanalysis, not a strength. The causes for those differences are undefined, you cannot assume they are due to “trends over time in cCD management and control”. Unless there is evidence to support the assumption, this should be moved to the limitations section and state that the underlying reasons for the differences between time categories is undefined/unknown. | Thank you for your comment. We’ve moved this to our limitations. |
| Reviewer #4: The results support the data. The method and limitations of the analyzes are clearly stated. The authors emphasized how the data would assist in understanding the topic. It is a study on public health and will contribute scientifically in this field. | Thank you for your comment! |
| **Editorial and Data Presentation Modifications?** | |
| Minor revision  The authors completed to satisfaction all the recommendations, except the following:  Line 46-47: should say: …….via oral consumption of contaminated food or beverage [ELIMINATE THIS "with triatomine feces"], and through vertical transmission from mother....Note: This is because food and beverages can be contaminated by other than triatomine feces, for instance crushed infected insects and anal secretions from infected marsupials.  Line 108-109: should say: ……….associated with cCD [ELIMINATE THIS: "infection"] were considered…..Note: This is because is congenital Chagas disease or T. cruzi congenital infection but not congenital Chagas disease infection | Thank you for your comment. We’ve removed “with triatomine feces” as well as “infection” |
| Reviewer #2: As above, a revision to limitations is needed. | Thank you for your comment, this has been revised. |
| Reviewer #4: Accept | Thank you! |
| **Summary and General Comments** | |
| Reviewer #2: No additional comments to this revision. | Thank you! |
